# Emphysematous aortitis: report of two cases and CT imaging findings

**DOI:** 10.1259/bjrcr.20170006

**Published:** 2017-04-06

**Authors:** Mohamad Syafeeq Faeez Md Noh, Anna Misyail Abdul Rashid, Aida AR, Norafida B, Yusri Mohammed, Ezamin A R

**Affiliations:** ^1^Department of Imaging, Faculty of Medicine and Health Sciences, Universiti Putra Malaysia, Seri Kembangan, Malaysia; ^2^Department of Medicine, Faculty of Medicine and Health Sciences, Universiti Putra Malaysia, Seri Kembangan, Malaysia; ^3^Department of Diagnostic Imaging, Hospital Serdang, Serdang, Malaysia

## Abstract

Emphysematous aortitis is a rare condition that manifests through the presence of air within or surrounding the inflamed aorta. Aortic inflammation may result from either an infective or a non-infective cause. Recognition of this rare condition is important as the resultant clinical deterioration may be rapid and lead to inevitable death. Cross-sectional imaging, with its relatively wide availability, proves an important tool in the diagnosis and subsequent management of this condition. We report two such cases encountered in our centre, with particular focus on the imaging findings on CT.

## Background

Aortitis refers to the inflammatory process involving the aortic wall, which can be either infective or non-infective in origin.^1^ The aorta, under normal conditions, is very resistant to infection. However, certain conditions may predispose the aorta to infection; these include atherosclerotic disease, a pre-existing aneurysm, diabetes and other immune-susceptible states and the presence of medical devices or open surgery.^1–3^ A wide spectrum of causative organisms may be found including* Staphylococcus aureus, Salmonella, Pneumococcus, Escherichia coli, Treponema pallidum, Candida, Aspergillus *and* Tuberculosis*.^1^ Prompt diagnosis is critical in treatment planning and instituting subsequent therapies. We describe the CT imaging findings of two patients encountered in our centre.

## Case reports

### Patient 1

**Figure 1.**
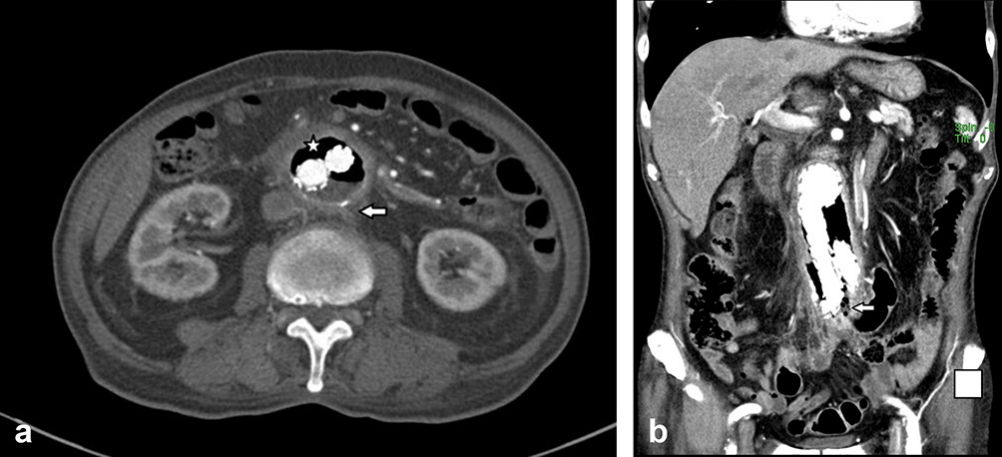
Contrast-enhanced CT images in axial (a) and coronal (b) sections: a—air is noted surrounding the endovascular graft (star), with aortic wall inflammation (arrow); b—air with multiple air pockets seen surrounding the endovascular graft, extending to the level of the common iliac arteries (arrow).

A 79-year-old male, with positive history of ischaemic heart disease and dyslipidemia, was referred to our centre for further management of an incidental finding of an abdominal aortic aneurysm (AAA). Initial CT angiography of the aorta revealed an infrarenal AAA measuring 6.3 cm × 7.0 cm × 20.0 cm, which extended to the level of the right common iliac artery. There were scattered wall calcifications. The thoracic aorta was normal. After discussing with the patient and family members, a decision was made to repair the AAA via an endovascular approach. A bifurcated endovascular stent graft was deployed via a percutaneous femoral route. The procedure was successful. However, 3 months post procedure, the patient came back with complaints of fever of 39°C, associated with chills, loss of appetite and back pain. He reported no vomiting or diarrhoea. Full blood count revealed a total white cell count of 15 × 10^9^ l^–1^ with predominant neutrophilia. In view of the underlying AAA, a repeat CT was pursued. This revealed the presence of air surrounding the endovascular graft, with associated inflammatory changes and aortic wall thickening. The air was seen extending to the proximal common iliac artery (Figure 1). Medical therapy and appropriate antibiotics were initiated, with a plan to embark on surgery once the patient was more stable. Blood cultures grew *Staphylococcus aureus*. Unfortunately, the patient succumbed to sepsis after 1 week of medical therapy.

### Patient 2

**Figure 2.**
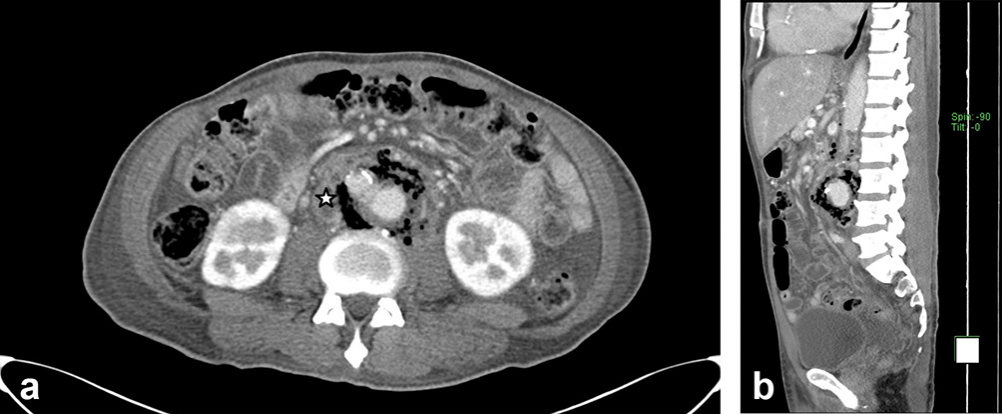
Contrast-enhanced CT images in axial (a) and sagittal (b) sections: a—periaortic collection (star) noted, with associated periaortic air pockets; b—an emphysematous region is noted, in close proximity to the vertebrae, with no evidence of bony destruction.

A 54-year-old male who was a chronic smoker and had long-standing heart failure was referred to our centre for investigation of abdominal pain with elevated liver enzymes. He reported with fever with recorded temperature of 38°C, with nausea, vomiting and diarrhoea. There were no chills and rigors. Full blood count showed a total white cell count of 20 × 10^9^ l^–1^ with predominant neutrophilia. A multiphasic CT hepatobiliary system was initially planned, with a provisional diagnosis of hepatobiliary pathology. During the image acquisition, incidental finding of an infrarenal AAA was noted. The AAA measured 3.4  cm × 3.4 cm × 2.7 cm with protruding mural thrombus. No clear fat plane was identified, and multiple air pockets were seen. Retroperitoneal and para-aortic collections were present, with no bony destruction (Figure 2). In view of the findings, blood cultures were taken and medical therapy instituted. The blood cultures came back positive for *Salmonella;* thus antibiotic therapy was commenced according to the sensitivity. However, after 14 days of hospitalization, the patient unfortunately succumbed to complications of exacerbated heart failure.

## Differential diagnoses

1. Non-infective causes of aortitis, including Takayasu’s arteritis, ankylosing spondylitis and giant cell arteritis
2. Tuberculous aortitis
3. Syphilitic aortitis

## Discussion

Osler, in the year 1885, was the first person to coin the term “mycotic aneurysm” in which he described mushroom-shaped infectious aneurysms associated with bacterial endocarditis.^4^ The prevalence of recognized primary infection of aortic aneurysms has been cited between 0.7% and 2.6%, the most commonly identified causative agent being *Salmonella spp*.^5–7^ Certain pathogens, namely *Clostridium septicum*, have been associated with the presence of infective aortitis, with a concomitant underlying malignancy. Several reports associate *Clostridium septicum* infective aortitis with colonic malignancy.^8–11^

Under normal circumstances, the aorta is resistant towards infection. However, certain conditions may predispose it to infection, including atherosclerotic disease, an underlying aneurysm, patients who are immune-compromised and the presence of a medical device or open surgery. A few postulations of the pathophysiology are (1) direct extension of a local infection, (2) traumatic contamination, (3) septic embolism from endocarditis and (4) haematogenous spread from a distant source of bacteremia.^2,4,12^ We postulate that traumatic contamination (due to deployment of the endovascular device) was what likely happened in our first case, whereas a haematogenous spread from a distant source was likely the source in the second case, taking into account the history and underlying conditions.

Cross-sectional imaging proves to be an indispensable tool in the diagnosis and further management of this condition. CT is widely available and has multiplanar as well as post processing capabilities. Patients with this condition are often systemically unwell; thus a short image acquisition time is preferred. Among the signs of infective aortitis on CT are aortic wall thickening, periaortic fluid, a rapidly enlarging aneurysm, gas formation and air pockets and vertebral body destruction. Periaortic asymmetric fat stranding or a periaortic soft tissue mass are present in 48% of cases, and may be the only early signs of infection.^2^ Periaortic gas and vessel wall emphysema indicate more advanced disease,^10^ and therefore a poor prognosis, as noted above. With the advent of antibiotics, most of these signs are not usually seen.^12^

The role of MR, despite being able to provide excellent soft tissue contrast and not involving ionizing radiation, is limited in the acute setting, which is how these patients usually present. The longer acquisition time, the relative preponderance to artefacts in relation to CT, and limited access make MR a less preferred cross-sectional imaging modality compared to CT. However, if access to MR and patient cooperation are not limiting factors, MR may be best to demonstrate aortic wall oedema, particularly in the early stages and when aortitis is highly suspected.^12^ In the later stages, with gas formation surrounding the infective process, CT is a better option.

Accurate clinical diagnosis remains a challenge to treating physicians, as often these patients present with non-specific clinical signs and symptoms,^1^ as was the case with our second patient. He was initially investigated for hepatobiliary pathology, only to be found to have emphysematous aortitis. The diagnosis in the first patient was aided by the knowledge that he had a previous endovascular procedure prior to presentation. However, the patient still succumbed as emphysematous aortitis is known to be a life-threatening complication after endovascular aortic repairs.^13^

The mainstay of treatment involves medical therapy including appropriate antibiotics, preferably according to the sensitivity of the pathogen isolated from blood cultures. This is followed by surgical resection of the infected aortic segment with in situ or extra-anatomic reconstruction.^2^ The goal of surgery is diagnostic confirmation, control of sepsis, control of haemorrhage in cases where rupture has occurred and reconstruction of the aortic vasculature.^12^

## Conclusions

The diagnosis of “emphysematous aortitis” is a loosely used term to denote the presence of air in association with aortitis. A multipronged approach that includes clinical, laboratory and radiological findings is needed in guiding the approach to patient treatment strategies. CT is an excellent tool in recognition and diagnosis of this condition. Conforming to the current European Society of Cardiology guidelines on the management and treatment of aortic diseases, CT and if permissible MR, should be used as first-line imaging techniques in suspected aortic infections.^14^ Vigilance and early recognition enable prompt diagnosis and treatment and may potentially prove to be life saving.

## Learning points

1. Emphysematous aortitis is a life-threatening condition, albeit rare these days; early recognition followed by appropriate management may be life saving.
2. Certain conditions predispose the aorta to infection; recognizing these allows early and accurate diagnosis.
3. Clinical presentation alone may be vague and fail to lead to an accurate diagnosis. Hence, the role of cross-sectional imaging (for example, CT) is of utmost importance.
4. Certain CT features, when recognized, enable the treating physician to diagnose and institute appropriate treatment/ operative intervention.

## Consent

Written informed consent for the case to be published (including images, case history and data) was obtained from the patient(s) for publication of this case report, including accompanying images.
